# Social connectedness and healthcare engagement among adults with disabilities: The role of living arrangement

**DOI:** 10.1371/journal.pone.0349802

**Published:** 2026-05-20

**Authors:** Hyun-Jun Kim, Natalie Turner, Brittany Jones-Cobb

**Affiliations:** School of Social Work, University of Washington, Seattle, Washington, United States of America; University of Udine: Universita degli Studi di Udine, ITALY

## Abstract

People with disabilities face significant healthcare engagement barriers, leading to increased risks of preventable conditions and mortality. This is particularly concerning for the quarter who live alone. Despite recognition of healthcare engagement as a social process, empirical understanding remains limited. This study examined how social connectedness predicts healthcare engagement, testing whether disability status and living arrangements moderate these associations. We analyzed data from 335 U.S. adults (ages 21–83) via an online survey. Healthcare engagement was assessed through three indicators: having a usual source of care, a personal doctor, and routine checkups. Social connectedness included intimate network size and informal health information network size. Using logistic regression, we tested main effects of social connectedness after controlling for covariates and three-way interactions (social connectedness × disability × living arrangements). Informal health information network size significantly predicted having a usual source of care and a personal doctor while intimate networks showed no main effects. A three-way interaction revealed that larger informal health information networks improved personal doctor access among adults without disabilities (regardless of living arrangements) and adults with disabilities living with others, but not among those with disabilities living alone. For routine checkups, larger intimate networks reduced utilization among adults with disabilities living alone. Social connectedness plays a conditional rather than universal role in healthcare engagement. Adults with disabilities living alone represent a particularly vulnerable population who do not benefit from social connectedness in accessing healthcare, highlighting the need for targeted interventions addressing their unique barriers.

## Introduction

Healthcare engagement, active participation in health and healthcare systems [1,2], is critical for maintaining long-term health and managing chronic conditions [2]. Yet, disparities in healthcare engagement persist, particularly among socially marginalized populations [3–5]. Adults with disabilities are disproportionately affected, with one in four lacking a usual healthcare provider and one in six forgoing routine checkups due to cost [6,7]. Barriers to accessing healthcare contribute to reduced preventive care [3,4,8], delayed or forgone care [9], and increased risk of preventable comorbidities and mortality [3,5]. Compounding these challenges, more than a quarter of people with disabilities live alone compared to about 10% of people without disabilities [10]. Living alone, while reflecting autonomy [11], can contribute to the disablement process, social isolation, and increased healthcare needs [12,13]. While prior research has identified structural and individual-level predictors of healthcare engagement, less is known about the role of social connectedness – the multidimensional experience of relatedness and belonging in informal and formal social networks in terms of their structure, function, and quality [14] – especially for vulnerable subgroups at higher risk of social isolation, such as adults with disabilities and those living alone [6]. Social connectedness may provide resources for facilitating healthcare engagement [15], yet few studies have examined whether its impact varies by disability status and living arrangements.

While lacking universal consensus in its operationalization, healthcare engagement commonly encompasses several interrelated key elements. A usual source of care represents the security of having a designated place to seek health services and promotes preventive care receipt, continuity of care, and chronic condition management. Having a personal doctor facilitates relational continuity and tailored care delivery, with individuals reporting higher satisfaction and improved ability to navigate referrals and coordinate care. Annual routine checkups serve as essential mechanisms for early detection and intervention, particularly vital for adults with disabilities who face elevated risks for comorbidities [3]. For this population, regular preventive care enables prevention of secondary conditions and ongoing monitoring of functional changes, making these elements of healthcare engagement crucial for maintaining optimal health outcomes and quality of life.

Research has extensively documented multi-level barriers to healthcare engagement, including life course factors, structural factors, health literacy as well as individual characteristics. Early life experiences, such as potentially traumatic events occurring in childhood, have been associated with increased barriers to healthcare in adulthood including lack of insurance, forgone healthcare [16] and higher rates of missed or canceled appointments [17]. Structural barriers to healthcare engagement include limited provider availability and accessibility [6]; accommodation issues (e.g., long wait times for visits, length of time spent waiting in the office) [6,18]; communication challenges [18]; transportation difficulties [6,18]; stigmatized perceptions of people with disability; insufficient provider training on disability [18]; and cost-related barriers including inadequate health insurance coverage [19,20]. Health literacy, including both individual competencies to access and evaluate health information and supportive environments for seeking and understanding health information, also positively influences healthcare engagement [21].

While healthcare engagement has been conceptualized as both an individual state and a social process [1], few empirical studies have examined the role of informal healthcare-related support networks as a mechanism facilitating healthcare access. This study addresses this gap by drawing from the Network Episode Model (NEM), which posits that health behaviors are embedded in social processes [22,23].‌‌ The NEM considers illness as an ongoing process where individuals go through various events while being influenced by the advice, support, and resistance they encounter from their formal (e.g., physicians,) and informal (e.g., friends, families) networks [23]. Social influence operates through multiple channels (informational, instrumental, and normative) within formal and informal networks [24]. Within the NEM, healthcare engagement and utilization are shaped by a complex pattern of interactions over time involving members from both formal and informal networks [22].

Networks can both facilitate and hinder access to healthcare, as decisions around healthcare are rarely made in isolation. Social networks can influence whether an individual seeks care, which options individuals consider, and how they evaluate alternatives [24,25]. Social networks can also provide information about healthcare systems, assist in identifying appropriate providers, or offer transportation to services [15]. As such, participation in pro-medical networks can facilitate early and consistent use of health services and validate help-seeking [22]. Conversely, a lack of support among an individual’s social network may negatively affect an individual’s ability to recognize health issues or seek treatment, and social network norms that discourage healthcare use can disincentivize individuals from seeking care [15].

Disability often involves functional limitations that may increase need for support from others to navigate physical, informational barriers as well as environmental and institutional barriers that restrict full participation in health systems. Thus, these facilitating and constraining roles of social networks take on particular significance for people with disabilities [26]. Social networks may support health-related behaviors that promote adherence to medical recommendations and reduce the use of high-cost healthcare services, such as hospitalizations. In a study of 120 adults with physical disabilities in Brazil, 89.2% reported that family and friends helped them access health services, most commonly by assisting with appointment scheduling and accompanying them to visits [27]. A systematic review on healthcare barriers for women with disabilities found that married women could rely on family income, enabling more consistent access to healthcare [28]. Conversely, insufficient social support limited access to services [27]. Lack of support and heightened loneliness have been linked to delayed medical care [29], underscoring how social connectedness affects both access and timeliness of care. Living alone, which may indicate reduced social support, has been associated with higher emergency department admission rates and increased inpatient costs [30,31], though it has also been associated with higher use of general practitioner services [31]. Marriage, by contrast, has been associated with lower use of inpatient and skilled nursing facility care [32], suggesting that co-residence may reduce reliance on intensive services. Still missing from this literature is an understanding of how specific network functions (e.g., health-related networks) influence healthcare engagement.

Guided by the NEM, this study tests the hypothesis that social connectedness (i.e., intimate networks and informal health information networks) positively predicts healthcare engagement (i.e., usual source of care, having a personal doctor, and routine preventive care utilization) after accounting for historical life course factors (i.e., childhood relationship quality), structural barriers (i.e., financial constraints and healthcare discrimination), and health literacy as well as background characteristics including chronic conditions. In addition, this study investigates whether disability status and living arrangement moderate the association between social connectedness and healthcare engagement, with the hypothesis that this relationship will be strongest among adults with disabilities living alone.

## Methods

### Data

This online survey sample of 335 adults aged 21–83 was recruited via Prolific, an online research participant recruitment platform, which has evidenced high-quality data, in terms of comprehension, attention, and honesty [33]. Data collection took place from February 8 to February 28, 2025, with a stratification goal by age, race/ethnicity, sexual orientation, gender identity, disability status, and living arrangements. To be eligible to participate in this study, participants had to be age 18 and older and reside in the U.S. Eligible participants were sent the study information and anonymous survey link by Prolific. The online survey included questions on barriers and facilitators of healthcare utilization and social networks, capturing both structural and functional aspects of participants’ social connectedness. Participants who completed the survey were given $10 to thank them for their time.

This study was determined to qualify for exempt human subjects research status by the University of Washington Human Subjects Division Institutional Review Board (IRB ID: STUDY00020730). As such, the survey cover letter served as an implied consent form and included all required consent elements.

### Measures

#### Dependent variables.

Our main dependent variable was healthcare engagement, measured using three separate dichotomous outcomes (1 = yes; 0 = no). First, aligned with previous research, [34] participants were asked if they had a usual source of care, that is, a place they typically go to when they are sick or need advice about their health. Second, participants were asked if they had a person they thought of as their personal doctor or healthcare provider. Finally, participants were asked if they had received a routine checkup in the past 12 months.

#### Independent variables.

Social connectedness was measured using aspects of the Social Network Roster [14,35] modified for online survey distribution. The Social Network Roster was designed to capture various dimensions of participants’ social connectedness and has been utilized in large-scale aging studies administered to participants across a wide range of cognitive and functional capacities [36,37]. The Social Network Roster used in this study first asked participants to list up to six individuals (adults age 18+) with whom they most often discussed topics they considered important. Participants were then asked about their relationship to each network member as well as functional characteristics of the relationship (e.g., how close they felt to each person, who they talked to about health needs, etc.). We included two measures of social connectedness related to social network functions: the size of intimate networks and the size of informal health information networks. To measure the intimate network size, we used the question, “Compared to the others you mentioned, how close do you feel to each person?” with response options “1) Not very close; 2) Somewhat close; 3) Very close; 4) Extremely close.” We counted the number of people in a participant’s social network marked very or extremely close (possible range: 0–6). To measure the size of informal health information networks, we used three items from the Social Network Roster. For each network member named, participants were asked whether they would talk with them 1) about information on health issues they were concerned with, such as health screening, illness or treatments; 2) when they needed help to understand health information; and 3) when seeking a place to get the healthcare they need. For each item, the total number of network members who provided that support was summed. The final value for informal health information network size was calculated as the average of these three totals, representing the average number of social network ties providing health-related informational support across the domains.

To measure childhood relationship quality, participants were asked about their relationship with their family or household members during the first 18 years of their life. Responses ranged from 0 (Very bad) to 10 (Very good) [38]. Financial barriers to healthcare were assessed using two items asking how often, in the past 12 months, participants had forgone needed medical care or reduced prescribed medication use due to cost. Individuals who endorsed either item were categorized as experiencing financial barriers to healthcare. Discrimination in healthcare was assessed using the 7-item Discrimination in Medical Settings scale [39]. Participants were asked seven questions on how often they experience discriminatory treatment when seeking care (e.g., You are treated with less courtesy than other people; You are treated with less respect than other people). Each item was rated on a scale from 1) Never to 5) Always. The final score reflects the average across the seven items. Finally, health literacy was measured using the mean score of eight items assessing health literacy (e.g., Find information on health issues that concern you, such as health screening, certain illnesses, or treatments; Understand what your doctor says to you). Participants rated each item on a scale of 0 = Very difficult to 3 = Very easy) [40].

#### Moderators.

Disability status was measured dichotomously, defined as having at least one functional difficulty in these six domains: hearing, vision, cognition, mobility, self-care, and independent activities of daily living (IADLs) [41]. Living alone was also measured dichotomously (1 = Living alone; 0 = Living with others).

#### Background characteristics.

Demographic measures included age in years, sex measured dichotomously (1 = Female; 0 = Male), sexual orientation (1 = gay, lesbian, bisexual, queer, and sexual diverse vs. 0 = heterosexual), gender identity (1 = transgender vs. 0 = cisgender), race (White, Black/African American, Asian, American Indian or Alaska Native, Other race, and Multiracial), and ethnicity (1 = Hispanic or Latinx vs. 0 = Other ethnic groups). We also measured the total number of chronic conditions reported by the participant and whether the participant had any form of health insurance (1 = Yes; 0 = No). Chronic conditions included diabetes; high blood pressure; arthritis; osteoporosis; dementia; Alzheimer’s disease; cancer; chronic back pain; stroke; heart attack; any heart disease including angina or congestive heart failure; asthma; emphysema, chronic bronchitis, or chronic obstructive pulmonary disease (COPD); HIV/AIDS; depression; anxiety; and other medical issues.

### Analytic plan

Analyses were conducted using STATA (Version 19.5; Stata Corp, College Station, Texas). First, we estimated descriptive statistics to summarize the sample characteristics and study variables. We also assessed bivariate correlations to examine associations between independent variables, covariates (age, sex, number of chronic conditions, health insurance coverage), and the outcomes. Then, we estimated a series of multiple variable logistic regression models for each of the three outcomes. The first model included covariates and key predictors: living alone status, disability status, childhood relationship quality, financial barriers to healthcare, discrimination in healthcare, and health literacy. The second model included all variables from the first model and intimate network size and informal health information network size. The third model added interaction terms to the second model to test two three-way interaction effects: 1) intimate networks × disability status × living alone and 2) informal health information networks × disability status × living alone. All models used robust standard errors to improve the validity of inference and account for potential outliers given the small sample size [42].

## Results

### Sample characteristics

Demographic information and relationships between the study variables can be found in Table 1. The sample ranged in age from 21 to 83 was 60% female, 55% White, 21% Black/African American, 13% Asian, 2% American Indian or Alaska Native, 4% Other race, 5% Multiracial, 18% Hispanic or Latinx, 27% sexual minority, and 9% transgender individuals. Over 93% had health insurance coverage; 36% were living alone; and 35% had disability. Among those with disability, 41% were living alone. While a higher number of intimate networks was associated with a higher number of informal health information networks, both were significantly associated with health insurance coverage, lower likelihood of disability, better childhood relationship quality, higher health literacy, and the three healthcare engagement variables. A higher number of intimate networks was also associated with lower discrimination in health settings whereas the size of informal health information networks was not associated with healthcare discrimination. All healthcare engagement variables were also associated with older age, more chronic conditions, health insurance coverage, lower financial barriers, and higher health literacy. Usual source of care and having a personal doctor were associated with better childhood relationship quality, and having a personal doctor was associated with lower discrimination in health settings.

**Table 1:** Correlations and descriptive statistics for continuous and binary variables of interest.

	(1)	(2)	(3)	(4)	(5)	(6)	(7)	(8)	(9)	(10)	(11)	(12)	(13)	(14)	(15)
(1) Age (years)	1														
(2) Sex, female	.032	1													
(3) No. chronic conditions	.130*	.047	1												
(4) Health insurance coverage	.064	.003	.092	1											
(5) Living alone	−.073	−.063	.166**	−.102	1										
(6) Disability	−.036	.047	.435***	−.032	.070	1									
(7) Childhood relationship quality	.100	−.142*	−.264***	.132*	−.127*	−.301***	1								
(8) Financial barriers	−.117*	.108	.179**	−.260***	.084	.255***	−.240***	1							
(9) Discrimination in health settings	−.097	.079	.235***	−.151**	.044	.342***	−.323***	.390***	1						
(10) Health literacy	.152**	.035	.035	.117*	.008	−.140*	.173**	−.276***	−.366***	1					
(11) No. intimate networks	.028	.076	−.052	.155**	−.104	−.156**	.249***	−.054	−.174**	.263***	1				
(12) No. informal health info. networks	.066	.031	−.047	.149**	−.074	−.151**	.196***	−.087	−.088	.168**	.638***	1			
(13) Usual source of care	.119*	.044	.202***	.247***	−.045	.042	.112*	−.144**	−.071	.282***	.166**	.182**	1		
(14) Having a personal doctor	.189**	.085	.183**	.341***	−.069	−.008	.121*	−.220***	−.116*	.241***	.207***	.236***	.504***	1	
(15) Annual routine checkup	.188**	.057	.221***	.319***	−.037	.014	.074	−.204***	−.086	.175**	.187**	.147**	.451***	.516***	1
Mean (SD) or %	52.75 (11.53)	59.57	2.25 (1.77)	93.43	36.12	35.24	6.52 (2.97)	38.74	0.93 (0.81)	2.06 (0.51)	3.09 (1.65)	2.81 (1.65)	76.12	74.25	74.85
Range	21–83	---	0–8	---	---	---	0–10	---	0–4	0–3	0–6	0–6	---	---	---

Notes: SD = standard deviation; * p < .05; ** p < .01; *** p < .001

### Predictors of healthcare engagement

#### Usual source of care.

Table 2 presents the hierarchical regression results for having a usual source of care. Model 1 identified four significant predictors: number of chronic conditions (OR = 1.49, p = .001), health insurance coverage (OR = 3.85, p = .026), childhood relationship quality (OR = 1.13, p = .029), and health literacy (OR = 4.48, p < .001). The addition of social connectedness variables in Model 2 revealed that while informal health information networks showed a positive association with having a usual source of care (OR = 1.29, p = .045), intimate networks demonstrated no significant relationship. The final model (Model 3) tested three-way interactions among living arrangement, disability status, and social connectedness measures; none of these interaction terms reached statistical significance, suggesting these moderating relationships do not contribute to predicting usual source of care beyond the main effects identified in Models 1 and 2.

**Table 2:** Hierarchical binary logistic regression model predicting usual source of care (N = 317).

	Model 1		Model 2		Model 3	
OR	p	OR	p	OR	p
Age (years)	1.01	.491	1.01	.438	1.01	.447
Sex, female	1.36	.296	1.34	.319	1.39	.273
Number of chronic conditions	1.49	.001	1.46	.001	1.42	.004
Health insurance coverage	3.85	.026	3.51	.032	4.26	.013
Living alone	0.91	.759	0.95	.861	0.52	.546
Disability	1.40	.319	1.63	.159	3.31	.234
Childhood relationship quality	1.13	.029	1.13	.036	1.14	.039
Financial barriers	0.67	.227	0.71	.317	0.69	.285
Discrimination in health settings	1.24	.330	1.22	.380	1.24	.368
Health literacy	4.48	.000	4.46	.000	5.14	.000
Size of intimate networks	---	---	0.99	.939	1.07	.700
Size of informal health information networks	---	---	1.29	.045	1.09	.665
Interaction terms						
Live alone × Intimate networks	---	---	---	---	0.99	.956
Live alone × Disability	---	---	---	---	0.80	.884
Disability × Intimate networks	---	---	---	---	0.85	.743
Live alone × Disability × Intimate networks	---	---	---	---	0.75	.612
Live alone × Informal health information networks	---	---	---	---	1.49	.276
Disability × Informal health information networks	---	---	---	---	1.08	.838
Live alone × Disability × Informal health information networks	---	---	---	---	1.00	.999
	Wald chi2	p	Wald chi2	p	Wald chi2	p
	42.69	.000	5.59	.061	6.22	.515

Notes: OR = odds ratio; The analytic sample (N = 317) is smaller than the full sample (N = 335) due to listwise deletion of cases with missing data on select variables (missing rate < 2%).

#### Having a personal doctor.

**Fig 1:**
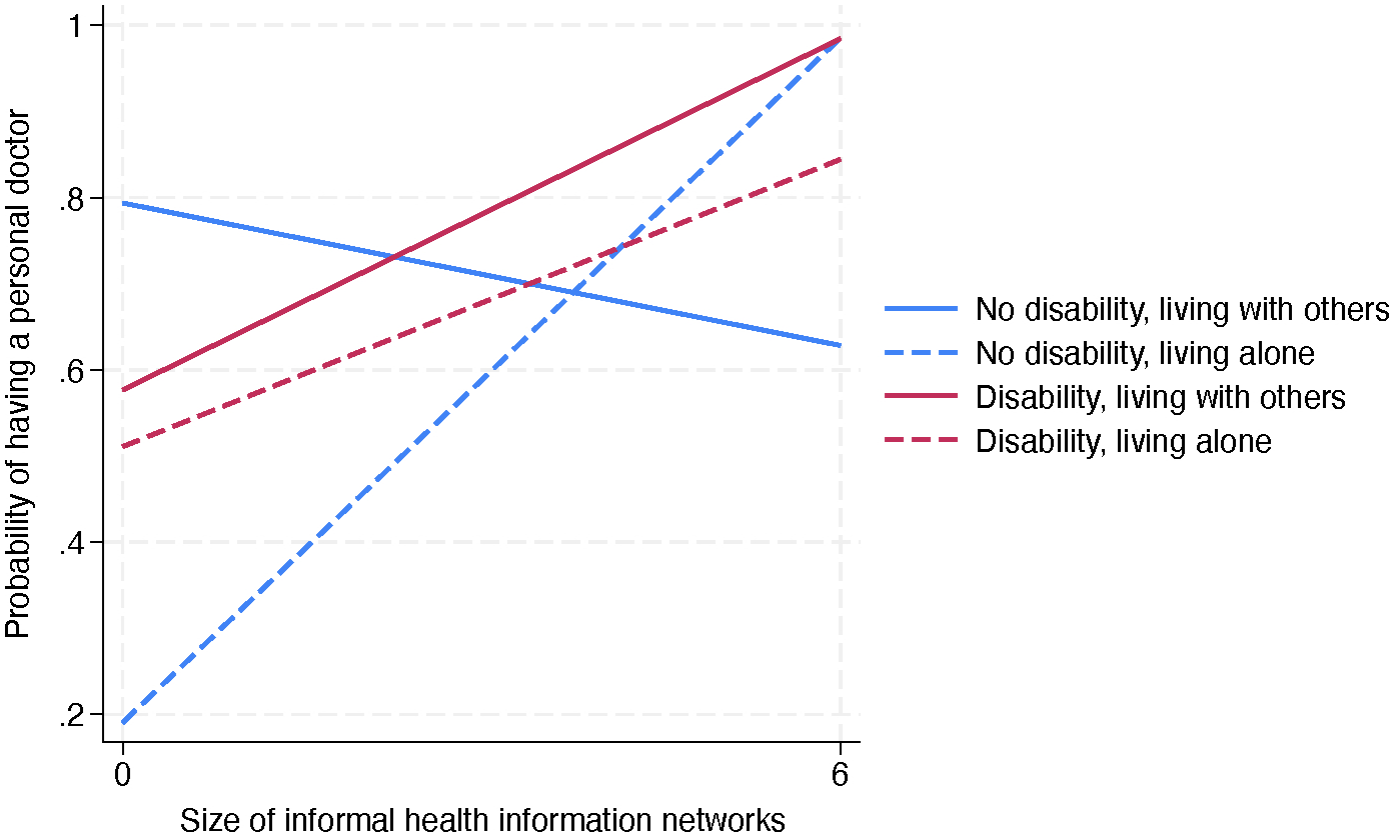
Moderated effect of size of informal health information networks on having a personal doctor by disability status and living arrangement. Slopes: No disability, living with others = −0.03, p = .363; No disability, living alone = 0.14, p < .001; Disability, living with others = 0.07, p =.006; Disability, living alone = 0.06, p =.178.

According to Model 1, as shown in Table 3, older age (OR=1.03, p = .023), greater number of chronic conditions (OR=1.41, p = .003), health insurance coverage (OR=10.14, p < .001), and higher health literacy (OR=2.28, p = .007) were associated with higher odds of having a personal doctor while financial barriers showed a negative association (OR=0.51, p = .043). Model 2 added social connectedness indicators, revealing informal health information networks as positively associated (OR=1.38, p = .023). Size of intimate networks did not predict having a personal doctor. Model 3 demonstrated significant two-way interactions: living alone × disability (OR=105.86, p = .001), living alone × healthcare networks (OR=4.30, p < .001), and disability × healthcare networks (OR=3.20, p = .006), along with a significant three-way interaction living alone × disability × healthcare networks (OR=0.13, p < .001). When no social connectedness was available, disability was a moderator between living arrangement and having a personal doctor: living alone, when compared to those living with others, was associated with a lower likelihood of having a personal doctor whereas living arrangement and having a personal doctor did not relate to each other. The three-way interaction indicates that informal health information networks only improve personal doctor access for individuals without disabilities living alone (slope = 0.14, p < .001) or individuals with disabilities living with others (slope = 0.07, p = .006) (Fig 1). Adults with disabilities living alone (slope = 0.06, p = .178) and living with others without disabilities (slope = −0.03, p = .363) did not show a statistically significant association between healthcare support networks and personal doctor access.

**Table 3:** Hierarchical binary logistic regression model predicting of having a personal doctor (N = 316).

	Model 1		Model 2		Model 3	
OR	p	OR	p	OR	p
Age (years)	1.03	.023	1.03	.017	1.03	.021
Sex, female	1.72	.069	1.70	.083	1.81	.074
Number of chronic conditions	1.41	.003	1.38	.004	1.41	.002
Health insurance coverage	10.14	.000	8.75	.001	20.13	.000
Living alone	0.83	.528	0.87	.649	0.11	.022
Disability	1.14	.725	1.39	.378	0.14	.044
Childhood relationship quality	1.09	.146	1.08	.238	1.07	.326
Financial barriers	0.51	.043	0.52	.060	0.49	.060
Discrimination in health settings	1.11	.641	1.06	.800	1.08	.763
Health literacy	2.28	.007	2.13	.015	2.46	.010
Size of intimate networks	---	---	1.03	.841	1.32	.162
Size of informal health information networks	---	---	1.38	.023	0.82	.375
Interaction terms						
Live alone × Intimate networks	---	---	---	---	0.62	.085
Live alone × Disability	---	---	---	---	105.86	.001
Disability × Intimate networks	---	---	---	---	1.17	.712
Live alone × Disability × Intimate networks	---	---	---	---	0.66	.451
Live alone × Informal health information networks	---	---	---	---	4.30	.000
Disability × Informal health information networks	---	---	---	---	3.20	.006
Live alone × Disability × Informal health information networks	---	---	---	---	0.13	.000
	Wald chi2	p	Wald chi2	p	Wald chi2	p
	42.23	.000	9.38	.009	28.35	.000

Notes: OR = odds ratio; The analytic sample (N = 316) is smaller than the full sample (N = 335) due to listwise deletion of cases with missing data on select variables (missing rate < 2%).

#### Annual routine checkup.

**Fig 2:**
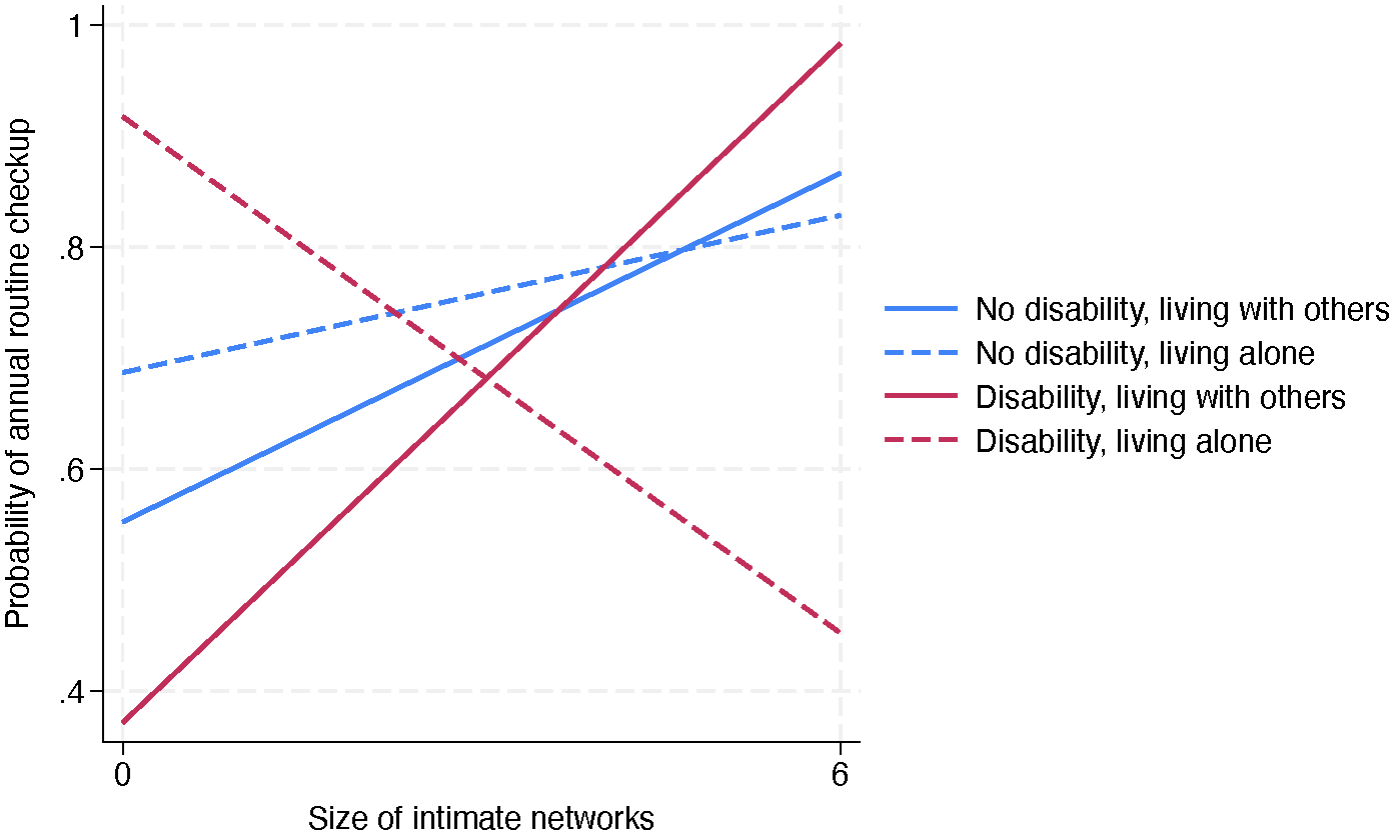
Moderated effect of size of intimate network on annual routine checkup by disability status and living arrangement. Slopes: No disability, living with others = 0.05, p =.035; No disability, living alone = 0.02, p =.422; Disability, living with others = 0.09, p =.011; Disability, living alone = -0.08, p =.034.

The hierarchical regression models for annual routine checkups (Table 4) showed significant positive associations with older age (OR=1.03, p < .05 across models), greater number of chronic conditions (OR=1.48, p = .001 for Model 1; OR=1.46, p = .001 for Model 2), and health insurance coverage (OR=7.27, p = .002 for Model 1; OR=6.31, p = .003 for Model 2) while financial barriers showed negative associations (OR=0.44, p = .015 for Model 1; OR=0.41, p = .011 for Model 2). While Model 2 revealed no significant effects of social connectedness, Model 3 identified one significant three-way interaction: living alone × disability × intimate networks (OR=0.24, p = .021), indicating that, for adults with disabilities living alone (slope = −0.08, p = .034), those with larger intimate networks had a reduced likelihood of annual routine checkup whereas for adults living with others without disabilities there was a positive association between routine checkup and intimate network size (Fig 2). No other interactions reached significance.

**Table 4:** Hierarchical binary logistic regression model predicting of annual routine checkup (N = 316).

	Model 1		Model 2		Model 3	
OR	p	OR	p	OR	p
Age (years)	1.03	.046	1.03	.033	1.03	.039
Sex, female	1.34	.327	1.29	.405	1.33	.380
Number of chronic conditions	1.48	.001	1.46	.001	1.53	.000
Health insurance coverage	7.27	.002	6.31	.003	7.45	.001
Living alone	0.90	.754	0.97	.937	0.86	.878
Disability	1.13	.740	1.22	.580	0.29	.271
Childhood relationship quality	1.04	.518	1.02	.802	1.00	.946
Financial barriers	0.44	.015	0.41	.011	0.36	.009
Discrimination in health settings	0.94	.781	0.94	.778	0.92	.734
Health literacy	1.45	.245	1.27	.461	1.34	.423
Size of intimate networks	---	---	1.21	.114	1.42	.038
Size of informal health information networks	---	---	1.01	.948	0.76	.174
Interaction terms						
Live alone × Intimate networks	---	---	---	---	0.83	.506
Live alone × Disability	---	---	---	---	4.00	.361
Disability × Intimate networks	---	---	---	---	1.81	.187
Live alone × Disability × Intimate networks	---	---	---	---	0.24	.021
Live alone × Informal health information networks	---	---	---	---	1.35	.372
Disability × Informal health information networks	---	---	---	---	1.11	.813
Live alone × Disability × Informal health information networks	---	---	---	---	2.29	.216
	Wald chi2	p	Wald chi2	p	Wald chi2	p
	36.07	.000	3.80	.149	12.70	.080

Notes: OR = odds ratio; The analytic sample (N = 316) is smaller than the full sample (N = 335) due to listwise deletion of cases with missing data on select variables (missing rate < 2%).

## Discussion

Despite the recognized importance of healthcare engagement for health-related quality of life, significant disparities persist, disproportionately affecting socially marginalized groups such as adults with disabilities and those living alone. While prior research has identified numerous structural and individual barriers, the role of social connectedness as a potential facilitator remains less understood. Guided by the NEM, which posits that health behaviors are shaped within social processes [22,23], this study sought to address this gap. We had two primary aims: first, to investigate whether two forms of social connectedness, intimate networks and informal health information networks, predict key indicators of healthcare engagement after accounting for life course, structural, and health literacy factors; and second, to test whether these relationships are moderated by disability status and living arrangements. Our findings confirm that social connectedness matters, but in complex and contingent ways. While informal health information networks were generally associated with better engagement, effects were not uniform. Crucially, the benefits of social connectedness were shaped by disability status and living situation. Most notably, for adults with disabilities living alone who are a group facing a potential double burden of marginalization, social networks sometimes failed to confer an advantage and, in one case, were associated with reduced engagement, challenging the presumption that social ties are universally beneficial.

The positive association between the size of informal health information networks and having both a usual source of care and a personal doctor aligns with the NEM [22] and prior research [15]. This finding suggests that these networks can facilitate healthcare engagement through distinct yet interconnected pathways. They provide crucial instrumental support, such as assistance navigating healthcare systems, finding providers, and scheduling appointments. Concurrently, they offer informational support through the sharing of practical advice, such as provider recommendations or guidance on navigating insurance systems. The contrast with the non-significant findings for intimate networks suggests that general emotional closeness, while valuable for overall well-being, is not sufficient to drive these logistically complex health behaviors [43].

One of the most critical findings of this study is the significant moderating role of disability status and living arrangements, which reveals a “double jeopardy” for those with a disability and living alone. Notably, informal health information networks improved access to a personal doctor for individuals without disabilities living alone and for those with disabilities living with others. In contrast, this informational health support provided no significant benefit for adults with disabilities living alone and cohabiting adults without disabilities. Those cohabiting without disabilities may have low healthcare engagement demand. For adults with disabilities living alone, compounding barriers (e.g., structural, financial, and accessibility-related) [6,20] may be too influential for informational support alone to overcome. While these individuals may have sizeable social networks, such networks may lack the immediate, instrumental resources that co-residence provides, such as a household member who can offer timely transportation or intensive in-person advocacy.

Another unexpected finding, which appears counterintuitive, is that larger intimate networks were associated with a reduced likelihood of annual routine checkups for adults with disabilities living alone. This is particularly striking when compared to the positive association for adults with disabilities living with others. This complexity challenges the prevailing “more support is always better” claim and demands nuanced interpretation. A couple of non-mutually exclusive mechanisms could explain this paradox. First, fully interpreting this finding requires knowing about the type, quality, and function of the intimate networks for disabled individuals living alone. Friend versus kin networks and proximal versus distant or virtual networks confer different support for health tasks [43,44]. Second, for this vulnerable group, the emotional and practical demands of maintaining close non-cohabiting networks [45] may conflict with limited energy and the resources necessary for proactive health tasks like scheduling and attending checkups [6]. Finally, while non-cohabiting networks provide companionship, they may lack health-specific competence or even hold attitudes that discourage “unnecessary” doctor visits [15,22]. This discouragement of doctor visits could be understood through the intersecting lenses of disability and autonomous living. Given that living alone often represents a hard-won achievement of independence [11], intimate networks may consciously or unconsciously reinforce this valued autonomy in ways that become a barrier to preventive healthcare. This finding accentuates a core tenet of the NEM that social networks are not merely conduits of resources but can also function as sources of obligation or norms that constrain health-seeking behavior [22].

The results also echo the well-established role of key structural and enabling factors in shaping healthcare engagement. The positive associations of health insurance coverage, a greater number of chronic conditions, and higher health literacy with the outcomes align with prior research [1,19,20], underscoring that social connectedness operates within a framework of these fundamental determinants and cannot compensate for their absence. Meanwhile, the negative relationship between financial barriers and annual routine checkups underscores that economic hardship remains a distinct deterrent to preventive services.

The findings of this study inform the application of the NEM for adults living with disabilities. The type and role of network, distinguishing intimate ties from health-specific ones, and the intersectionality of disability status and living arrangement are essential factors to incorporate. Consequently, future research should move beyond simple metrics of network size to investigate the composition, specific functions, and qualitative nature of social ties, with focus on vulnerable subgroups. These theoretical insights necessitate a reorientation of clinical practice. Healthcare systems should routinely screen for informal social resources to assess the functional capacity of a patient’s support system and identify patients at elevated risk for disengagement. It is also critical to develop formal support in partnership with disability organizations and to address structural barriers, such as transportation, cost, and clinic accessibility, directly, rather than assuming social networks can compensate for them. This highlights the importance of training providers to identify a patient’s social context as a key factor influencing healthcare engagement.

Several limitations of this study should be considered. First, the cross-sectional design precludes any causal inferences. While the NEM posits that social networks influence health behaviors, the reverse may be plausible. For instance, individuals more engaged with healthcare may have more opportunities to build health-specific social connectedness [43]. The counterintuitive finding that larger intimate networks were associated with reduced routine checkups for adults with disabilities living alone suggests the need for longitudinal research to test temporality. Second, the sample, though stratified for diversity, is not generalizable to a larger population. Participants were required to have internet access and be Prolific users, which likely excludes some of the most marginalized individuals, such as those facing disparities in digital literacy. This selection bias may mean our findings underestimate the true extent of barriers or the severity of social isolation faced by these vulnerable subgroups. Third, all data were self-reported, which is subject to recall and social desirability biases. Because disability status in particular was assessed via self-report and participants were recruited through an online platform, those with more profound cognitive or sensory impairments were likely underrepresented, which may limit the generalizability of findings to these subgroups. Furthermore, key variables were operationalized as dichotomous measures. The dichotomization of disability status and living arrangements, while useful for initial analysis, obscures considerable within-group heterogeneity. For example, “living alone” does not capture the quality or frequency of contact with non-cohabitant support networks. Finally, usual source of care was conceptualized with a single question, without reference to the expertise or reliability of that care, highlighting the need for further, more nuanced research regarding healthcare engagement.

## Conclusion

This study elucidates that healthcare engagement is a complex social process, revealing that its benefits can differ by disability status and living arrangements. Our findings reveal a “double jeopardy” for adults with disabilities living alone, for whom informational support failed to secure a personal doctor and larger intimate networks paradoxically reduced the odds of a routine checkup, challenging the presumption that social ties are beneficial across within-group diversity. Achieving equitable healthcare engagement may require a shift towards targeted, structural solutions. We must invest in formalized support infrastructures that are designed to directly address the compounded barriers adults with disabilities living alone face beyond merely fostering social connectedness.

## Supporting information

S1 Data Data dictionary. (XLSX)

S2 Data Data. (CSV)
